# Efficacy of a short-term residential smoking cessation therapy versus standard outpatient group therapy (‘START-Study’): study protocol of a randomized controlled trial

**DOI:** 10.1186/s13063-020-04253-x

**Published:** 2020-06-23

**Authors:** Jonas Dickreuter, Claudia Schmoor, Jürgen Bengel, Andreas Jähne, Jens A. Leifert

**Affiliations:** 1 Comprehensive Cancer Center, Prevention Team CMPT, University Medical Center Freiburg, Elsässerstraße 2, 79110 Freiburg, Germany; 2 Clinical Trials Unit, Faculty of Medicine and Medical Center, University Medical Center Freiburg, Elsässerstraße 2, 79110 Freiburg, Germany; 3 Department of Rehabilitation Psychology and Psychotherapy, Institute of Psychology, Albert-Ludwigs-University Freiburg, Engelberger Straße 41, 79085 Freiburg, Germany; 4 Rhein-Jura Klinik, Schneckenhalde 13, 79713 Bad Säckingen, Germany; 5 Breisgau-Klinik, Herbert-Hellmann-Allee 37, 79189 Bad Krozingen, Germany

**Keywords:** Smoking cessation, Residential, Inpatient, Behavioral therapy, Outpatient group therapy, Motivational interviewing, Prevention, Randomized controlled trial

## Abstract

**Background:**

In Germany, evidence-based outpatient smoking cessation therapies are widely available. Long-term abstinence rates, however, are limited. Studies suggest that short-term residential therapy enables a higher level of environmental control, more intense contact and greater support among patients and from therapists, which could result in higher abstinence rates. The aim of the current START-study is to investigate the long-term efficacy of a short-term residential therapy exclusively for smoking cessation, conducted by a mobile team of expert therapists.

**Methods:**

A randomized controlled trial (RCT) is conducted to examine the efficacy of residential behavior therapeutic smoking cessation therapy compared to standard outpatient behavior therapeutic smoking cessation group therapy. Adult smokers consuming 10 or more cigarettes per day, who are willing to stop smoking, are randomized in a ratio of 1:1 between therapy groups. The primary endpoint is sustained abstinence for 6-month and 12-month periods. Secondary endpoints include smoking status after therapy, 7-day point abstinence after the 6-month and 12-month follow-ups, level of physical dependence, cost-effectiveness, use of nicotine replacement products, health-related quality of life, self-efficacy expectation for tobacco abstinence, motivational and volitional determinants of behavior change, self-reported depressive symptom severity, adverse events and possible side effects. Assessments will take place at baseline, post-therapy, and at 6-month and 12-month intervals after smoking cessation.

**Discussion:**

There is a high demand for long-term effective smoking cessation therapies. This study represents the first prospective RCT to examine the long-term efficacy of a residential smoking cessation therapy program compared to standard outpatient group therapy as an active control condition. The residential therapeutic concept may serve as a new model to substantially enhance future cessation therapies and improve the understanding of therapeutic impact factors on tobacco abstinence. Utilizing a mobile team, the model could be applied efficiently to medical centers that do not have permanent and trained personnel for smoking cessation at their disposal.

**Trial registration:**

German Register for Clinical Trials (Deutsches Register für Klinische Studien), DRKS00013466. Retrospectively registered on 1 April 2019. https://www.drks.de/drks_web/navigate.do?navigationId=start.

## Administrative information

Note: numbers in curly brackets in this protocol refer to Standard Protocol Items: Recommendations for Interventional Trials (SPIRIT) checklist item numbers. The order of the items has been modified to group similar items (see http://www.equator-network.org/reporting-guidelines/spirit-2013-statement-defining-standard-protocol-items-for-clinical-trials/).

**Table:** 

Title {1}	Efficacy of a short-term residential smoking cessation therapy versus standard outpatient group therapy (‘START-Study’): study protocol of a randomized controlled trial
Trial registration {2a and 2b}	German Register for Clinical Trials (Deutsches Register für Klinische Studien), DRKS00013466
Protocol version {3}	Protocol Version 1.2 from 9 October 2019
Funding {4}	The trial was funded by the German Cancer Aid (Deutsche Krebshilfe e. V.), a charitable organization for cancer prevention and treatment (Project number: 70112396)
Author details {5a}	^1^Comprehensive Cancer Center, Prevention Team CMPT, University Medical Center Freiburg, Elsässerstraße 2, 79110 Freiburg, Germany. ^2^Clinical Trials Unit, Faculty of Medicine and Medical Center, University Medical Center Freiburg, Elsässerstraße 2, 79110 Freiburg, Germany. ^3^Department of Rehabilitation Psychology and Psychotherapy, Institute of Psychology, Albert-Ludwigs-University Freiburg, Engelberger Straße 41, 79085 Freiburg, Germany. ^4^Rhein-Jura Klinik, Schneckenhalde 13, 79713 Bad Säckingen, Germany. ^5^Breisgau-Klinik, Herbert-Hellmann-Allee 37, 79189 Bad Krozingen, Germany. JL, AJ and CS designed the study. JL is responsible for trial management. JL and AJ obtained funding for the study, JL conducted the pilot study. JD drafted the manuscript, and is responsible for the diagnostic assessments. CS is responsible for the statistical planning and analysis. JD is in charge of the additional statistical analyses. JL, JB and CS revised the manuscript. All authors read and approved the final manuscript
Name and contact information for the trial sponsor {5b}	German Cancer Aid (Deutsche Krebshilfe e. V.), Buschstraße 32, 53113 Bonn, Germany. Correspondence: Deputy division director of funding, Annika Marks, annika.marks@krebshilfe.de
Role of sponsor {5c}	This funding source had no role in the design of this study or in writing the manuscript and will not have any role during its execution, collection, management, analyses, or interpretation of the data, or decision to submit results.

## Introduction

### Background and rationale {6a}

Tobacco consumption is one of the leading risk factors for early death and disability worldwide [1]. Continued smoking is regarded as a major risk factor in the development of severe medical conditions, such as cancer [2], pulmonary disease [3] and cardiovascular disease [4, 5]. Moreover, due to productivity loss and high follow-up treatment costs, it imposes a heavy burden on the economy [6]. Successful smoking cessation, on the other hand, can have a significantly positive impact on life expectancy [7, 8] and quality of life [9], even after decades of smoking.

Research shows that the majority of smokers wish to reduce (30%) or even quit (59–68%) smoking [10, 11]. However, nicotine dependence is a complex clinical disorder, which often takes a chronic course [12]. Therefore, spontaneous unassisted quit attempts are common but not very promising: 60% of unassisted quit attempts fail within the first 2 weeks, 81% suffer a relapse within the first month and 95% suffer a relapse within the first year of abstinence [13–15]. In contrast, a combination of evidence-based pharmacotherapeutic and behavioral therapeutic interventions for smoking cessation is advised [15–17] and was proven effective in a large-scale Cochrane review in 2014 [18]. However, evidence for long-term effectiveness is relatively low. Even with a combination of different evidence-based approaches for smoking cessation, only up to 20% of smokers achieve abstinence over a period of at least 6 months [19].

In an effort to increase the long-term efficacy of smoking cessation for strong addictions, the American Society of Addiction Medicine (ASAM) developed an algorithm to intensify procedures for smoking cessation from unassisted self-help over counseling and outpatient group therapy (combined with medication) to a residential therapy away from the patient’s daily environment and habits [20]. It can be assumed that high-intensity residential smoking cessation therapy has several advantages over interventions in outpatient contexts [21]. Most smokers live in environments that contain a wide range of behavioral cues for smoking and triggers associated with nicotine consumption. Residential smoking cessation therapies enable increased environmental control and a cue-free environment, which is especially crucial to prevent relapses within the first 24 h of smoking cessation [22–24]. In addition, the residential setting enables high-frequency smoking cessation therapy and complementary supportive interventions from a multiprofessional team, which could be important to establish a new day structure and provide the opportunity for learning and testing novel skills to facilitate long-term abstinence (cf. [25–27]). Complementary interventions like physical activity and relaxation techniques can further cease craving as well as other nicotine withdrawal symptoms [27–29]. The close support from therapists and the various activities within the patient group foster group cohesion and could improve the sense of social support, which is an important factor in the initiation and maintenance of abstinence [30].

Empirical studies on residential smoking cessation predominantly focus on brief counselling or highly selective groups to which patients with primary health issues other than smoking are admitted. Thus, resources to support smoking cessation during residential therapy are mostly limited to one brief counselling appointment, ranging from 5 min to 2 h, self-help materials and supplementary follow-up calls after discharge. For a detailed overview, see Kazemzadeh et al. and Rigotti et al. [31, 32]. Different scientific reviews have shown brief counseling interventions to be cost-effective as first-line therapy for smokers, yet long-term abstinence rates remain rather low [26, 31–35]. There is reason to doubt that a brief therapy session allows for enough support of motivational and volitional processes as well as development and evaluation of new behavioral patterns for long-term abstinence. In accordance with evidence-based outpatient therapy, residential therapy sessions should also take at least several hours to adequately incorporate an abstinence decision, planning, environmental control, risk assessment and relapse prevention (cf. [36, 37]). Only a few published studies have focused on more extensive residential smoking cessation therapies. These relate to uncontrolled or cohort studies with limited methodological quality. However, outcomes suggest significantly increased 6-month or 12-month abstinence rates from 42.6 to 64.7% [21, 38–43]. In a large American cohort study with 226 residential smokers, Hays et al*.* [21] reported significantly higher 6-month abstinence rates of 52% from 8-day residential smoking cessation therapy directly compared to 27% in outpatient therapy with 4327 patients, conducted from the same research group. It should be noted, however, that the total time provided for smoking cessation therapy in the inpatient group largely exceeded that in the outpatient group, which could have led to a strong dose–response effect [21]. These results are consistent with evidence from our uncontrolled pilot study that found 6-month abstinence rates of 50% for a residential smoking cessation therapy [44]. Comparatively high abstinence rates of up to 12 months have been reported in an American study with a 7-day residential smoking cessation (57% [40]) and in studies from Austria implementing a 21-day residential format (63.3% and 42.6% in studies with heavily dependent smokers [42, 43]), but with all studies missing control groups. Lastly, a 5-day stepwise residential nicotine cessation program has shown satisfactory abstinence rates of 31% at follow-up exceeding 2 years, even when counting participants who could not be reached as smoking [41]. In Germany, the vast majority of evidence-based therapeutic smoking cessation is conducted in an outpatient setting on a weekly basis over the course of 3–8 weeks. Scientific evaluations of residential therapy for smoking cessation are missing completely.

In conclusion, preliminary evidence suggests that residential smoking cessation therapies contain specific characteristics that could significantly increase abstinence rates. Yet the effectiveness of comprehensive smoking cessation therapy in a residential setting has to be determined in a scientific study design. To date, there are no data available from randomized controlled trials on the efficacy of residential therapy exclusively for smoking cessation. To bridge this gap, we are conducting a randomized controlled trial on the long-term efficacy of a newly designed short-term residential therapy with intensive provision of therapeutic modules, exclusively for smoking cessation. Considering the large number of evidence-based outpatient smoking cessation therapies in Germany, we are carrying out a comparative efficacy trial of residential therapy against the outpatient standard therapy. Depending on the outcome, this therapeutic concept may serve as a new format which can be applied to other medical centers without trained personnel. Furthermore, the new model might substantially influence future cessation therapies and provide deeper insight into intrapersonal determinants (e.g. self-efficacy expectation) and their interaction in smoking cessation.

### Objectives {7}

The aim of the current START-study (‘Vergleich Stationärer mit Ambulanter Raucherentwöhnungs-Therapie’) is to determine whether and to what extent the efficacy of behavioral therapy-based smoking cessation can be increased by a 9-day, time-limited intensive therapy program in a residential setting (superiority trial). We hypothesize that a residential smoking cessation therapy program is superior in terms of abstinence rates in comparison to an outpatient control condition. Moreover, we expect that the residential condition will result in a better health-related quality of life, and may lead to an improvement in cost efficiency when compared to an outpatient control condition. Lastly, we will evaluate whether changes in motivational and volitional variables to stop smoking will predict abstinence rates at the 12-month follow-up. Potential negative effects with regard to withdrawal symptoms and side effects from stop-smoking aids and other adverse events will be systematically evaluated.

### Trial design {8}

**Fig. 1:**
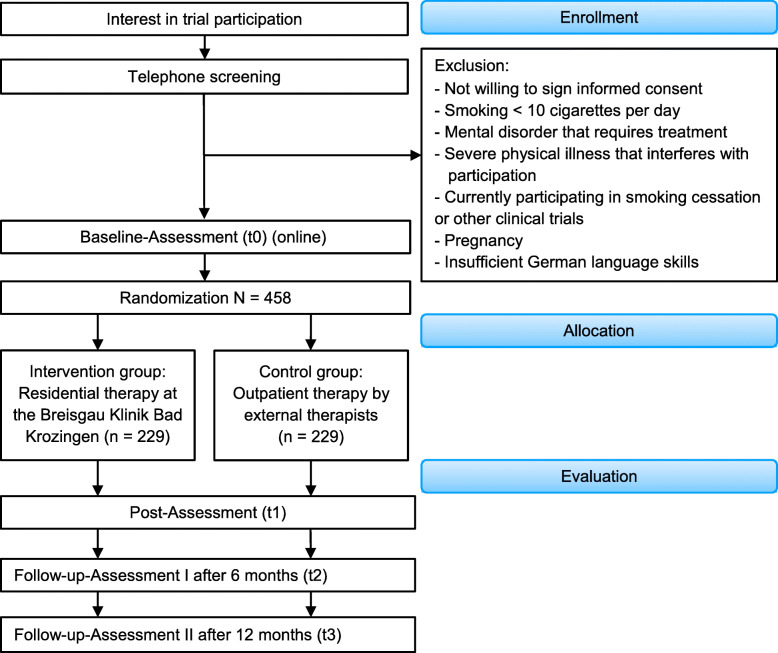
Flow chart of inclusion and study procedure

In a single-center prospective randomized two-arm control group design (RCT), we examine the efficacy of brief residential smoking cessation group therapy compared to existing evidence-based outpatient smoking cessation provided for 6 weeks on a weekly basis. The study is based on a pilot study which investigated the feasibility of the study design [44]. Self-report and observer-rated assessments will take place at baseline (t0) prior to randomization, post-therapy (t1), at a 6-month follow-up (t2) and at a 12-month follow-up (t3) (for a detailed overview of the procedure, see Fig. 1). The study is conducted in accordance with the standards of ethical practice as approved by the University of Freiburg ethics committee (No. EK-Freiburg 583/17).

## Methods: participants, interventions and outcomes

### Study setting {9}

Participants can apply from anywhere in Germany and are randomized to either the residential therapy group at the Breisgau Klinik, a rehabilitation clinic in Bad Krozingen, in the southwest of Germany, or to the outpatient therapy group, which is conducted as close as possible to the participant’s place of residence.

### Eligibility criteria {10}

Adult smokers consuming at least 10 cigarettes per day, who are willing to stop smoking, are screened for eligibility. Prospectively, we do not include participants with: at-risk levels of alcohol consumption (according to the German Head Office for Dependency Matters [45]); illegal substance abuse; mental disorders that require therapeutic treatment (meaning patients with severe mental illness, not on stable medical conditions, in ongoing psychotherapy as well as in inpatient treatment); severe physical illness that interferes with participation in the study, especially diseases with a life expectancy of less than 12 months; participation in other smoking cessation therapy programs or clinical studies during the intervention period (including hypnosis, acupuncture and phone counseling); pregnancy; or insufficient German language skills.

### Who will take informed consent? {26a}

Study applicants are informed about the purpose and eventual risks of the trial and screened for eligibility by telephone. In the event of a positive screening, they then receive detailed written information about the study procedures, a declaration of consent, a data protection agreement and the questionnaire for baseline assessment. Information includes the voluntary basis of participation and the option to withdraw from the study at any time without any negative consequences. Informed consent and consent to data protection are obtained separately from all participants. Participants who meet all inclusion criteria and none of the exclusion criteria are asked to provide written consent and to complete the baseline assessment that is included in the study.

### Additional consent provisions for collection and use of participant data and biological specimens {26b}

In a supplementary information sheet, participants are informed about their data protection access rights, rectification, cancellation and opposition to data collection in accordance with the General Data Protection Regulations of the EU. Participants are also asked for permission by the research team to share relevant data with staff from the University Medical Center Freiburg, the Breisgau Klinik Bad Krozingen and corresponding outpatient smoking cessation therapists. Consent for the provision of a urine sample for analysis is obtained from the telephone screening and by written consent.

### Interventions

#### Explanation for the choice of comparators {6b}

According to the S3 guidelines of the AWMF, behavior therapeutic groups possess the highest level of evidence and are thus strongly recommended as treatments for smoking cessation [16]. In order to assess the effect of conducting these groups within a short period in a residential setting, abstinence rates are compared to evidence-based weekly outpatient standard group therapies for smoking cessation. Both conditions are based on the same therapeutic rationale and have a similar total duration of therapeutic sessions, but vary in the frequency of therapeutic sessions and complementary supportive offers (such as relaxation techniques).

##### Intervention group

A residential behavioral group therapy program consisting of 10–12 participants is conducted over a 9-day period at the Breisgau Klinik. The methodical basis for this smoking cessation therapy is motivational interviewing, as described by Miller et al. [46]. This comprises the evidence-based behavioral smoking cessation therapy ‘Rauchfrei in 6 Wochen’ [37], which includes: preparation for abstinence (information about physical and psychological functionality of tobacco dependence, dealing with craving and withdrawal symptoms, motivational support to make a reasoned decision to quit smoking); the termination of tobacco consumption in the group (stimulus control, training of behavioral alternatives); and relapse prevention (adopting health-promoting behavior, relaxation training, social support, dealing with risk factors and relapses). It is flanked by supportive activities such as therapeutic exercise, relaxation techniques and nutrition counseling. Six sessions of smoking cessation therapy, lasting 1.5 h each, are performed only by trained and certified therapists of the Comprehensive Cancer Center Freiburg (CCCF), University Medical Center Freiburg. Supportive interventions are provided by qualified personnel belonging to the Breisgau Klinik. Beyond the smoking cessation sessions, participants take part in planned activities and general routines established by the rehabilitation clinic. In accordance with scientific recommendations [17] and present clinical guidelines for outpatient smoking cessation [16], participants receive advice on the use of stop-smoking aids and licensed medication to alleviate withdrawal symptoms. Use of stop-smoking aids and medication is voluntary. To increase treatment adherence, participants in the residential group are required to pay a study contribution of €50.

##### Control group

The comparative group refers to evidence-based outpatient behavior therapeutic smoking cessation. It is located close to the participant’s place of residence or workplace and provided on a weekly basis with a common therapy duration of 1.5 h per week over a 6-week period (consisting of at least 9 h in total of group therapy) by external therapists. In singular cases, the outpatient therapy course is tightened to three therapy sessions over the course of 3 weeks, lasting 3 h each, which is accepted on condition of similar content, extent and total duration. Outpatient therapists are recruited from the nationwide database of BZgA (Bundeszentrale für gesundheitliche Aufklärung), which is based on the qualification of the conducted program and the therapist. Standardized quality assessments of the therapeutic rationales and the framework of provision (e.g. group size, costs, recommendations of stop-smoking aids) are conducted to ensure treatment integrity and high comparability to the residential therapeutic procedures. Supportive offers within the outpatient smoking cessation group beyond the behavior therapeutic content (e.g. acupuncture) are permitted and documented for evaluation. In Germany, outpatient smoking cessation is in part refunded by public health insurance. In order to ensure similar financial conditions between both groups, participants in the outpatient group receive a study compensation of an additional €75, which results in approximately €50 in final costs for each participant. Study compensation is granted after written confirmation of the attendance by the therapists. Cooperating outpatient therapists receive compensation of €30 per study participant for their expenses (screening and confirmation of participants’ attendance).

#### Criteria for discontinuing or modifying allocated interventions {11b}

The feasibility of the study design and the recruitment procedures have been evaluated and proven successful in two separate pilot trials for both therapeutic groups. Predefined stopping criteria included insufficient study participation and sample response rates for biochemical verification of the smoking status. No further criteria for modifications or abortion criteria for the main study have been defined.

#### Strategies to improve adherence to interventions {11c}

All participants receive detailed written information regarding the dates and conditions of the smoking cessation program and are reminded 1 week prior to the commencement of their therapies in order to improve uptake rates. Furthermore, financial compensation, or in the case of the residential group, financial contribution is expected to increase commitment to participation. The number of therapy sessions is documented separately by both participants and therapists. Participants receive financial compensation to incentivize the return of urine samples for biomedical verification, resulting in increased return rates.

#### Relevant concomitant care permitted or prohibited during the trial {11d}

To avoid biased results, participants must state that they are not currently undergoing or intending to undergo psychotherapy and will not participate in any other clinical study or smoking cessation therapy throughout the course of the study. Use of health-care services and smoking cessation therapies outside the study after the end of the therapy program will be assessed at the 12-month follow-up.

#### Provisions for post-trial care {30}

Post-trial care is not intended: no specific risks have been reported regarding participation in residential or outpatient smoking cessation therapies [40, 42, 47]. Both treatment groups have access to standard healthcare treatment.

### Outcomes {12}

#### Primary and secondary endpoints

**Fig. 2:**
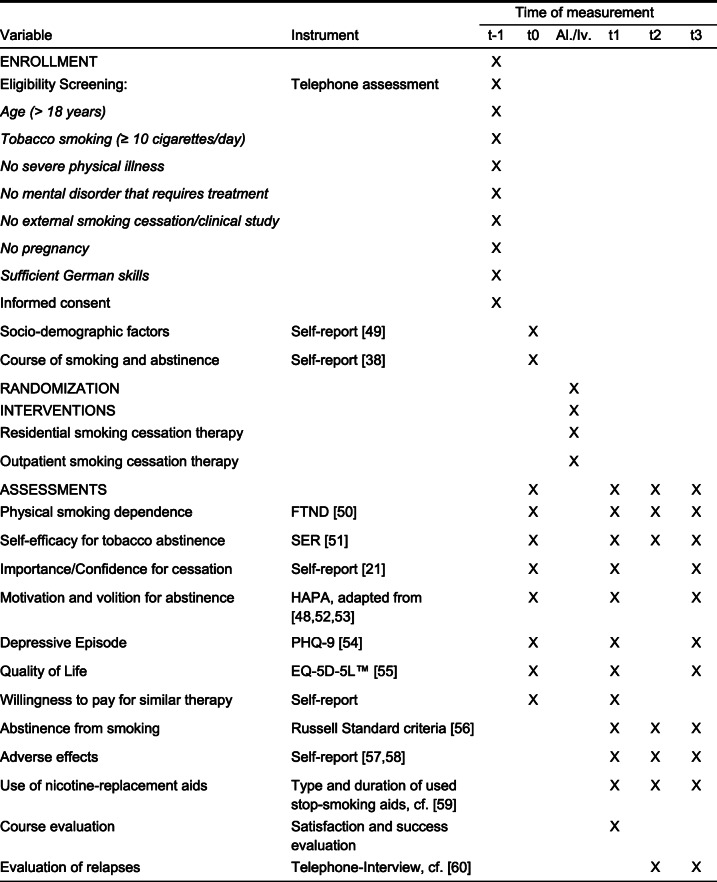
SPIRIT schedule of enrollment, interventions, and assessments. Abbreviations.* t-1* enrollment, *t0* baseline-assessment, *Al./Iv.* allocation/intervention, *t1* post-assessment after completion of therapy, *t2* 6-month follow-up-assessment, *t3* 12-month follow-up-assessment, *FTND* Fagerström Test for Nicotine Dependence, *SER* Selbstwirksamkeits-Skala zur Raucherentwöhnung (engl. self-efficacy scale for smoking abstinence), *HAPA* Health Action Process Approach, *EQ-5D-5L* EuroQol 5-dimensions questionnaire for the assessment of health-related quality of life [21, 38, 48–60]

The primary endpoint is the continuous abstinence (6-month and 12-month periods of abstinence) of participants following residential smoking cessation therapy, compared to the outpatient control group (see [18]). The secondary endpoints are the 7-day point abstinence, smoking status after the end of therapy, level of physical dependence, cost-effectiveness and amount of willingness to pay for a similar residential or outpatient therapy, motivational and volitional variables according to the HAPA model [48], self-efficacy expectation for tobacco abstinence, reasons for and degree of relapses as well as adverse events, such as depression (for a detailed overview, see Fig. 2).

### Measures

#### Primary endpoint

The 6-month and 12-month continued smoking abstinence will be assessed in accordance with international Russell Standard criteria via categorical questions [56]: participants will be classified as abstinent if they smoke no more than five cigarettes during the observation period. In an intention-to-treat analysis, participants lost to follow-up will be counted as relapsed. A recent multicenter trial has shown that, after smoking cessations, there is a high risk of participants inflating their abstinence rates in self-reports when compared to biochemical verification [61]. Hence, abstinence will be validated by urine cotinine assessments at the 12-month follow-up. Urine cotinine is the most widely used biomarker in smokers as it is more sensitive than blood cotinine, non-invasive [62, 63] and can specifically distinguish between first-hand and second-hand tobacco exposure [64].

##### Tobacco abstinence

The point prevalence of smoking status after the therapy as well as the 7-day abstinence at 6-month and 12-month follow-up intervals will be assessed for additional comparisons.

##### Physical smoking dependence

The self-reported level of physical nicotine addiction will be assessed with the Fagerström Test for Nicotine Dependence (FTND [50]). The FTND is an internationally used economic questionnaire and comprises six items, including the quantity of cigarettes smoked and the degree of dependence or, rather, interference with daily routine. The sum score of four dichotomous items from 0 to 1 and two multiple choice items from 0 to 3 indicates the level of physical dependence from none or weak (0–2), medium (3–4), and strong (5–6) to very strong (7–10) physical dependence. The FTND has been shown to be predictive of successful smoking cessation; the higher the level of dependence, the lower the rate of abstinence [65].

##### Aids for abstinence

Intake and period of usage of different kinds of stop-smoking aids as supporting agents for tobacco abstinence will be investigated following therapy, after the 6-month and 12-month follow-ups. Assessments include medications authorized in Germany, such as nicotine patches, gums, sprays, sublingual tablets or other inhalants as well as bupropion (Zyban) and vareniclin (Champix). Additionally, the use of e-cigarettes will be evaluated. Results from a recent study suggest that e-cigarettes could significantly help in quitting the smoking of tobacco. In contrast, caution is advised because the long-term use of e-cigarettes indicates an addiction shift rather than actual abstinence from smoking [59]. Due to a lack of information on long-term health risks and new reports on vaping-induced lung injury, e-cigarettes are not primarily advised as a stop-smoking aid [66]. Use of e-cigarettes is critically discussed as a means of harm reduction, based on preliminary evidence available for either of the therapies [67]. We will assess the use of e-cigarettes and conduct analyses of this subgroup of participants in comparison to those using other stop-smoking aids.

##### Course evaluation

As measures for acceptance of the therapy, numbers of outpatient sessions attended will be assessed by the participants and by the therapists, separately. Reasons for discontinuation of therapy will be recorded. Satisfaction with the intervention will be evaluated on a 5-point Likert scale ranging from ‘not at all satisfied’ to ‘fully satisfied’. Therapeutic success will be evaluated on a 5-point Likert scale from ‘very low therapeutic success or condition worsened’ to ‘very good therapeutic success or condition much improved’. Furthermore, participants will be asked whether they would recommend the therapy with a dichotomous answer format (‘yes/no’).

##### The self-efficacy expectation for tobacco abstinence

Confidence to cope with typical high-risk situations for smoking will be assessed with the ‘Selbstwirksamkeits-Skala zur Rauchentwöhnung’ (SER [51]), the German short form of the Smoking Abstinence Self-Efficacy Scale [68]. The scale consists of nine items that are rated on a 5-point Likert scale ranging from 1 (‘not at all confident’) to 5 (‘very confident’) to generate an overall sum. A higher total score indicates higher self-efficacy expectation. The SER showed excellent internal consistency (α = 0.95) and a high retest reliability (*r* = 0.85 [51]).

##### Quality of life

Health-related quality of life will be assessed with the five-level German version of the EQ-5D (EQ-5D-5L [55]). This is one of the most commonly used instruments in general health assessment, available in more than 130 languages, and can be used for clinical and economical health evaluation [69]. The EQ-5D-5L consists of a descriptive system containing five dimensions (mobility, self-care, usual activities, pain/discomfort and anxiety/depression) and a visual analog scale to indicate the respondents’ overall state of health from ‘the best health you can imagine’ to ‘the worst health you can imagine’. The descriptive system is rated on a 5-point scale ranging from no problems to slight, moderate, severe and extreme problems [55]. The psychometric properties of the EQ-5D-5L have not been evaluated in a smoking sample to this date. However, the EQ-5D-5L has shown it can discriminate between smokers and non-smokers and can detect changes in quality of life after smoking cessation [70, 71].

##### Cost-effectiveness analysis

To evaluate efficacy from an economic perspective, therapy costs and subjective health gains will be compared between both groups. Indices for perceived health improvement on an individual level will be quality-adjusted life years (QALYs) calculated with the EQ-5D-5L [72, 73] at baseline and at the 12-month follow-up. Direct costs for the outpatient therapy and the residential therapy will be assessed; travel expenses will be estimated from the distance to the intervention locations and the type of transport. Incremental cost-effectiveness ratios (ICERs) are defined as costs per QALY gained [74]. Germany does not use a general willingness to pay threshold for cost-effectiveness (cf. the USA with $50,000 per QALY [75]). Therefore, willingness to pay for the therapy will be assessed directly from the participant’s perspective.

##### Motivation and volition

Very little is known about the processes that mediate successful smoking cessation [76–78]. Yet evidence points to the relevance of motivational and volitional processes [77]. To gain a deeper understanding of the intrapersonal processes necessary to gain and maintain abstinence from smoking, different stages of intention formulation and intention implementation will be assessed, based on the HAPA model [48]. To our knowledge, no standardized questionnaire for the assessment of the HAPA in smoking behavior has so far been published. Thus, items were adapted and translated from other HAPA studies on smoking behavior [48, 52, 53] as well as the recommendations of Sniehotta et al. [79] for measuring lifestyle change. Twelve items were generated to assess the processes of risk perception, smoking-specific self-efficacy expectation, outcome expectancies, intention to stop smoking, action planning, coping planning and action control to stop smoking. One item was designed to measure risk perception under social perspective: ‘Compared to people your age and gender, how would you estimate the likelihood that you will develop severe health problems (e.g. pulmonary diseases, cardiovascular diseases or cancer)’. This is rated on a 4-point Likert scale, ranging from 0 (‘much lower’) to 3 (‘much higher’) (see [48]). The other 11 items have been formulated as individual statements (e.g. ‘If I quit smoking, my health will improve.’) and rated on a 4-point Likert scale ranging from 0 (completely disagree) to 3 (fully agree). Items will be assessed at baseline and at the post-therapy and 12-month follow-up.

##### Adverse effects

Depressive mood and adverse events are assessed in order to record potential negative treatment effects and reasons for relapse. Residential smoking cessation programs have been shown to be safe in various contexts. Although no particular adverse effects of smoking cessation provided in a residential setting have been reported [40, 42], nicotine withdrawal symptoms [57] and adverse effects of therapy will be carefully protocolled, such as deterioration of mood and sleep.

Self-reported depressive symptoms will be collected with the PHQ-9 [54], the 9-item depression module from the Patient Health Questionnaire [80], at baseline, post-therapy and 12-month follow-up. The PHQ-9 assesses the frequency of adverse symptoms within the last 2 weeks on a scale from 0 (‘not at all’) to 3 (‘nearly every day’). The sum score ranges from 0 to 27, with cutoff points of 5 for mild, 10 for moderate, 15 for moderately severe and 20 for severe depression [81, 82]. Indications of suicidal ideation are covered in the last item of the questionnaire (values > 0) and will be monitored at each point of assessment. Participants who affirm having had suicidal thoughts or thoughts of self-harm will be examined over the telephone by trained psychologists for risk assessment. Interviews will be protocolled. Despite its brevity, the PHQ-9 has a high reliability (strong internal consistency between α = 0.79 and 0.89 [80, 81]) and a high validity (comparable sensitivity and specificity to other depression questionnaires [82]).

According to currently available scientific literature, further adverse events such as irritability, aggressiveness, dysphoric mood, insomnia and increased appetite or weight gain will be monitored as well as side effects of the stop-smoking medication, such as vomiting, nausea and skin irritation of the nicotine patch (cf. [57, 58]). Lastly, reasons and dates of relapses will be assessed to identify high-risk situations which might not have been covered in one of the therapy groups. Likewise, number of days smoking as well as number and duration of attempts to regain abstinence will be evaluated.

### Participant timeline {13}

Please see Fig. 2 for the assessment overview.

### Sample size {14}

The sample size calculation is based on nicotine abstinence after 6 months as the primary endpoint of the trial. According to Hays et al. [21, 38] and clinical experiences in cooperation with our local network for smoking cessation, 6-month abstinence rates of ≈ 27% from outpatient smoking cessation under routine conditions are expected. At a two-sided significance level of α = 5%, the null hypothesis of equality of abstinence probabilities in both groups should be rejected with a power of 90%, if the residential program increases the abstinence probability to 50%. To date, no data on dropout rates for prospective controlled studies on residential smoking cessation are available. Therefore, estimations of dropout rates are based on published studies on outpatient behavioral therapy smoking cessation groups. At a conservative estimate, therapy-uptake dropout after randomization will be 40% (cf. [83], 31% (*n* = 139) [84]; 34% (*n* = 406)). Additionally, studies suggest a loss to follow-up rate after starting the therapy of 20% ([85], 5% (*n* = 558) [86]; 6% (*n* = 1041) [87]; 14% (*n* = 2707) [88]; 19% (*n* = 220)). Thus, we expect a total dropout rate of 52%. The primary analysis will be conducted according to the intention-to-treat principle including participants who started the smoking cessation program. For this analysis, participants whose smoking status cannot be assessed will be classified as non-abstinent. Under these assumptions, observed abstinence rates are expected to be 22% versus 40%, respectively. Based on a two-sided χ^2^ test, a sample size of 137 participants per therapy group is necessary. Accounting for the assumed therapy-uptake dropout, a total of 458 participants (229 per group) will be randomized.

### Recruitment {15}

Participants are recruited throughout Germany with a diverse approach via networking therapists for smoking cessation and general practitioners, advertisements in local newspapers and on social networks, announcements in scientific journals, press releases from the University Clinic Freiburg and the Comprehensive Cancer Center Freiburg website.

### Assignment of interventions: allocation

#### Sequence generation {16a}

Participants are randomized between therapy groups in a ratio of 1:1. Block randomization with randomly varying block size is performed based on computer-generated lists. Block sizes are documented separately from the study protocol in a document that is not accessible to investigators.

#### Concealment mechanism {16b}

Randomization is performed centrally at the Clinical Trials Unit in order to guarantee therapy allocation confidentiality. If a participant has given his or her written consent, then the participant’s identification code, year of birth, sex and confirmation that inclusion/exclusion criteria are fulfilled will be sent by fax to the central randomization office at the Clinical Trials Unit for randomization. To maximize therapy-uptake rates, all participants are screened and subsequently randomized as close as possible to the available therapy dates.

#### Implementation {16c}

The allocation sequence was generated by a statistician of the Clinical Trials Unit not involved in study planning or conduct. After receiving the randomization results from the Clinical Trials Unit, the investigators inform the participants about their group assignment by telephone and provide information regarding admission into their respective therapy groups.

### Assignment of interventions: blinding

#### Who will be blinded {17a}

Given the characteristics of the study, the therapists who conduct the smoking cessation therapies cannot be blinded to the study condition. Biochemical verification of the abstinence status will be conducted blind to the randomization arm. Data analysts have access to the trial database and, therefore, are not blinded. The statistical methods for the final analysis were prespecified.

#### Procedure for unblinding if needed {17b}

Not applicable due to an unblinded study design.

### Data collection and management

#### Plans for assessment and collection of outcomes {18a}

Self-report data will be collected using standardized questionnaires in a secure encrypted online-based assessment system. Standardized observer-based assessments will be conducted via telephone by trained interviewers.

#### Plans to promote participant retention and complete follow-up {18b}

To maximize retention, participants receive financial reimbursement as well as a reminder of therapy commencement and are contacted by telephone and email for all assessments. Moreover, telephone interviews are scheduled, if possible, and participants can choose from different methods of completing the questionnaires.

Participants may withdraw from the study for any reason at any time. Reasons for dropout will be protocolled, if possible.

#### Data management {19}

All relevant study data are collected on electronic CRFs (eCRF) using the system REDCap (Research Electronic Data Capture, https://www.project-redcap.org/), browser-based electronic data capture software.

The electronic forms were developed and validated for completeness and correctness of formats, branching logic and edit checks by the data management team at the Clinical Trials Unit. A user acceptance test was performed by the CCCF study team.

Data entry is done by CCCF study staff. Additionally, participants can choose to fill out their questionnaires either on paper or directly into the eCRF. Access to the eCRF is regulated by personal data entry user profiles with limited rights for CCCF study staff, and directly by the personal email address entered in a special master file form for the participants. Participants’ access is managed by CCCF study staff and is limited to the respective periods of time.

Data entry and data corrections on e-forms are automatically tracked in the audit trial created by the eCRF system. All data collected during the trial are entered as timely as possible. Reports and standard dashboards assist CCCF study staff in monitoring progress of data entry. The survey tool supports reminders for participants who do not fill out their questionnaires in a timely manner.

#### Confidentiality {27}

Personal data necessary for conducting the study are shared only with the prevention team of the University Medical Center Freiburg that conducts the study. All online assessments are protected using an encrypted connection to prevent unauthorized access by third parties. Data for the statistical evaluation will be pseudonymized and electronically processed for analysis, which will be carried out by staff of the University Medical Center*.* All data obtained are handled in accordance with the legal data protection regulations and the guidelines of the University of Freiburg ethics committee. In line with the recommendations of the data-protection supervisor of the University Medical Center Freiburg and the *Guidelines for Safeguarding Good Scientific Practice* of the DFG, anonymized data will be archived for 10 years on a secure institutional storage medium [89].

#### Plans for collection, laboratory evaluation and storage of biological specimens for genetic or molecular analysis in this trial/future use {33}

Cotinine values will be determined with liquid chromatography–mass spectrometry (LC-MS/MS) to validate the non-smoking status after 12 months. The procedure has been developed and validated by MVZ Clotten Laboratory Freiburg (Freiburg im Breisgau, Germany), which will analyze all samples. MVZ Clotten is not otherwise related to the trial. Urine sample kits including detailed instructions on collection and dispatch will be sent to all participants stating that they have stopped smoking. About 10 ml of urine will be collected from each participant and all samples will be sent immediately by post to MVZ Clotten. To avoid misclassification of abstainers and passive smokers as actively smoking, a cutoff value of 250 ng/ml urine will be used as the baseline to identify active smokers [90, 91]. Due to unclear scientific evidence, no adjustments for creatinine concentration will be made [92, 93]. Cotinine will be stored at − 80 °C for 6 months to allow for plausibility checks prior to disposal.

### Statistical methods

#### Statistical methods for primary and secondary outcomes {20a}

A logistic regression model will be used to assess the efficacy of the therapy on the primary endpoint of abstinence after 6 months. The therapy effect will be tested using the Wald test at a two-sided significance level of 5%, and as an estimate of the effect size, the odds ratios will be given with 95% confidence interval. If the hypothesis of no therapy effect on abstinence after 6 months could be rejected, based on the closed test procedure, a confirmatory test of the therapy effect on abstinence after 12 months will be performed at a two-sided significance level of 5% using the same analysis. Additionally, the following sensitivity analyses will be performed. These will be per-protocol analyses including only participants who have completed the entire therapy and provided data on their smoking status. Furthermore, missing data on smoking status will be handled using multiple imputation methods [94]. Due to possible non-random therapy-uptake dropout in both randomized groups, possible group differences will be accounted for in additional sensitivity analyses including all randomized participants regardless of therapy uptake. In this analysis, non-starters will be categorized as smokers. Based on the assumed abstinence probabilities and assumed minimum therapy-uptake rates, this analysis would lead to observed 6-month abstinence rates of 13% versus 24%. With 229 randomized participants per group, this leads to a power of 85%. Secondary analyses will be conducted to assess non-compliance. Comparisons involve participant baseline characteristics of dropouts (non-starters and dropouts after the start of therapy) and compliers.

#### Interim analyses {21b}

No interim analyses are planned. No elevated risks for participants are expected. Feasibility of the residential study design has been already confirmed in pilot trials [44].

#### Methods for additional analyses (e.g. subgroup analyses) {20b}

The prognostic effect of baseline characteristics (e.g. age, gender, socioeconomic status, smoking status prior to randomization, smoking-specific self-efficacy expectation) on smoking status and the predictive effect via interactions with the therapies will be evaluated in univariate and multivariate logistic regression models. If tests on interactive effects result in *p* < 0.05, the results will be presented in subgroups. No alpha adjustment for multiplicity in secondary analyses will be performed. Only the results of the primary endpoints abstinence after 6 and 12 months will be interpreted in a confirmatory manner. The *p* values from other analyses will be interpreted in a descriptive sense. All analyses will be conducted using SAS 9.4. The Clinical Trials Unit of the University Medical Center Freiburg will supervise data management and analyses.

#### Methods of analysis to handle protocol non-adherence and any statistical methods to handle missing data {20c}

These methods are referenced in Section {20a}.

#### Plans to give access to the full protocol, participant level-data and statistical code {31c}

These plans are referenced in Section {29}

### Oversight and monitoring

#### Composition of the coordinating center and trial steering committee {5d}

The coordinating center consists of the CCCF study team with the principal investigator and the study coordinator, as well as the Clinical Trials Unit, which is represented by the biometrician and the data manager. The principal investigator and the biometrician are responsible for the design of the START-study and prepare the study protocol and revisions jointly. Moreover, the principal investigator is responsible for the conduct of the study, the communication with all parties involved and the direction of publications and study reports. The Clinical Trials Unit conducts the randomization, and implements and supervises the eCRF. The biometrician and data manager are responsible for quality control of the data collection and for data administration and analysis, independently from the CCCF study team. The study coordinator is responsible for the adherence of the study team to the study protocol, and for monitoring the participant recruitment and data collection. Trained members of the study staff are in charge of participant recruitment and screening and conduct the residential therapy. Other members not personally involved in the residential therapy carry out the questionnaires and interviews. Data are entered by a research assistant of the study staff, who is not otherwise involved in conducting the study.

No steering committee is intended (see Section {21a}).

#### Composition of the data monitoring committee, its role and reporting structure {21a}

Not applicable: the applied interventions are based on non-invasive methods and do not contain any specific regulatory requirements or any increased risks for participants that require independent monitoring. Adverse events and indications of suicidal ideation are monitored directly by the study personnel.

#### Adverse event reporting and harms {22}

Special emphasis will be given to systematically evaluate potential negative effects of therapy, such as deterioration of the smoking status, depressive mood and worsening of health-related quality of life. Further adverse events, especially indications of suicidal ideation, are monitored for and managed by trained psychologists.

#### Frequency and plans for auditing trial conduct {23}

The trial conduct is reviewed on a weekly basis by the project management group. Monthly meetings of the trial steering group are held to monitor the trial conduct, randomization and data management according to the study protocol. A data monitoring committee was not considered as this is a low-risk intervention (see Section {21a}).

#### Plans for communicating important protocol amendments to relevant parties (e.g. trial participants, ethical committees) {25}

Protocol amendments will require consent from the principal investigator and the Clinical Trials Unit. Amendments that potentially affect ethical standards, the study design and the safety of participants or the scientific validity will be transparently reviewed and approved by the University of Freiburg ethics committee. All relevant parties will be informed. Amendments will be listed and described in the trial reports and the trial registry, protocol versions and dates will be updated.

### Dissemination plans {31a}

In addition to providing the anonymized data to the funding foundation, the results will be published in clinical trial registries and in international and national clinical journals (e.g. *Nicotine & Tobacco Research*, *Health Psychology*, *Sucht*) and presented at scientific conferences (e.g. European Conference on Tobacco or Health, Deutsche Konferenz für Tabakkontrolle). The key findings of the study will be released to all participants and the general public on the website of the University Medical Center Freiburg. The developed residential smoking cessation therapy design will be available for further research projects and provides the opportunity for national and international research collaborations.

## Discussion

An intensive residential program for smoking cessation could provide a great opportunity for participants to refrain from smoking in a short period of time in a controlled environment. This offers an increased level of support and enhanced learning opportunities to develop new behavioral routines. The smoking cessation therapists are employed independently of the rehabilitation clinic and could be deployed mostly autonomously from preexisting routines. Thus, the residential program could be easily applied in other contexts and medical centers. This study builds on several uncontrolled and cohort studies that indicate significantly higher abstinence rates from more intensive residential smoking cessation programs than can be expected from an outpatient therapy program [21, 38–44]. Before a residential model of smoking cessation should be recommended for use in routine care, randomized controlled data on long-term efficacy is needed. Based on a pilot study [44], this START-study will evaluate a new, brief residential smoking cessation group therapy program. It will be conducted by a mobile applicable team of therapists certified for smoking cessation. In a prospective RCT, the efficacy will be compared to a control group by a standard outpatient smoking group therapy program. Special emphasis will be given to systematically assess the long-term 6-month and 12-month efficacy and the motivational and volitional principles that moderate the outcomes.

This study will have some limitations. First, the required time and effort for participation (e.g. leave from work, distance and travel time to the therapies) might differ between participants and the therapeutic groups, which could affect motivation and lead to a selective therapy-uptake dropout. To minimize this potential bias, advantages and disadvantages of both therapies will be outlined equally and discussed during screening. Participants are only included in the study if they are able and willing to participate in both the residential therapy and the outpatient therapy. Second, the extent and procedures of the outpatient smoking cessation programs might differ due to a large number of different external therapists conducting the intervention. To ensure a high comparability between therapies, the search for outpatient therapies is based on the BZgA register to ensure therapeutic qualification and all therapeutic rationales and procedures are screened for eligibility based on prospectively defined criteria. The residential smoking cessation therapy is designed to match the amount and content of the outpatient therapy. Therapists are committed to a strict therapeutic rationale to ensure comparable conditions in both groups. To account for potential differences in the extent of additional supportive interventions in both groups, comparative cost-efficacy analyses will be computed. As another limitation, we have different types of stop-smoking medication including e-cigarettes. Due to the sample size we can only assess these moderators in an explorative approach. Finally, the study sample will be too small to test for potential interactive effects between subject characteristics and therapy success. Thus, findings on subgroups of participants who are especially likely to profit from this type of therapy or subgroups especially likely to experience negative effects will remain preliminary.

On the other hand, this study will be the first randomized controlled study to examine residential therapy in conditions that are close to routine care, compared to outpatient smoking cessation already implemented. Results are expected to yield high internal validity while allowing for conclusions about this new therapy model which can be generalized for other contexts to influence and improve future smoking cessation therapies.

## Trial status

This is Protocol Version 1.2 from December 4, 2019. Recruitment started on January 23, 2019. To date, 155 participants have been randomized and 106 participants have started one of the interventions. The estimated date for the completion of recruitment is September 2020. The analysis is expected to take place in 2021.

## Data Availability

After study completion, anonymous data will be archived for the long term on an encrypted institutional drive in accordance with the *Guidelines for Safeguarding Good Scientific Practice* [89]. Access to used and/or analyzed datasets of the current study will be made available from the trial sponsor on reasonable request.

## Abbreviations

ASAM: American Society of Addiction Medicine
DRKS: Deutsches Register für Klinische Studien
DFG: Deutsche Forschungsgemeinschaft
EQ-5D-5L: EuroQol 5-dimensions questionnaire for the assessment of health-related quality of life
FTND: Fagerström Test for Nicotine Dependence
HAPA: Health Action Process Approach
ICER: Incremental cost-effectiveness ratio
PHQ-9: Patient Health Questionnaire-9
QALY: Quality-adjusted life year
RCT: Randomized controlled trial
REDCap: Research Electronic Data Capture
START: STationäre versus Ambulante RaucherentwöhnungsTherapie
SER: Selbstwirksamkeits-Skala zur Raucherentwöhnung
WHO: World Health Organization
WMA: World Medical Association
